# The impact of a standardized vocal loading test on vocal fold oscillations

**DOI:** 10.1007/s00405-020-05791-5

**Published:** 2020-02-27

**Authors:** Matthias Echternach, Jamal Huseynov, Michael Döllinger, Manfred Nusseck, Bernhard Richter

**Affiliations:** 1 Division of Phoniatrics and Pediatric Audiology, Department of Otorhinolaryngology, Munich University Hospital (LMU), Campus Großhadern, Marchioninistraße 15, 81377 Munich, Germany; 2 Freiburg Institute of Musicians’ Medicine, Freiburg University, Freiburg, Germany; 3 Department of Otolaryngology/Head and Neck Surgery, Clinics of Villingen-Schwenningen, Villingen Schwenningen, Germany; 4 Division of Phoniatrics and Pediatric Audiology, Department of Otolaryngology Head and Neck Surgery, University Hospital Erlangen, Friedrich-Alexander-University Erlangen-Nürnberg, Erlangen, Germany; 5 Medical Faculty, Freiburg University, Freiburg, Germany

**Keywords:** Vocal loading, High speed imaging, Dysphonia severity index, Minimal intensity

## Abstract

**Introduction:**

Vocal loading capacity is an important aspect of vocal health and is measured using standardized vocal loading tests. However, it remains unclear how vocal fold oscillation patterns are influenced by a standardized vocal loading task.

**Methods:**

21 (10 male, 11 female) vocally healthy subjects were analyzed concerning the dysphonia severity index (DSI) and high speed videolaryngoscopy (HSV) on the vowel /i/ at a comfortable pitch and loudness before and after a standardized vocal loading test (10 min standardized text reading, at a level higher than 80 dB (A) measured at 30 cm from the mouth).

**Results:**

Changes in DSI were statistically significant, diminishing by 1.2 points after the vocal loading test, which was mainly caused by an increase of the minimum intensity. However, the pre-post comparison of HSV derived measures failed to show any statistically significant changes.

**Conclusion:**

It seems necessary to analyze the effects of a standardized vocal loading test on vocal fold oscillation patterns with respect to softest phonation and phonation threshold pressure rather than comfortable pitch and loudness.

Level of evidence

2c

## Introduction

Vocal loading capacity is an important aspect of vocal health and a reduction in such capacity might be a sign of dysphonia. Especially for voice professionals, such as teachers, priests, actors, singers or employees in call-centers [1] vocal loading capacity is of special interest and an impairment of the capacity might result in economic problems. However, in clinical circumstances, measurement of vocal loading is not easy to perform.

Studies using accelerometers have been used to estimate the vocal dose representative of realistic daily voicing activity [2–9]. In this context, it was shown that the dose during professional voice use differs from normal voice use outside working hours [8]. Furthermore, the background noise was found to differ during the day [8]. However, in a recent study of teachers, the Lombard effect [10], voice adaptation by means of higher sound pressure level (SPL) during increased background noise, was not verified for all subjects [11]. In addition, it was shown that the level of vocal dose might vary greatly inter-individually. Therefore, such measurements using accelerometers do not necessarily show the extent to which the vocal dose is related to vocal problems or signs of vocal fatigue.

To measure such a relation, vocal loading tests have previously been established for clinical voice measurement. Such tests involve the patient/subject voicing at a minimum sound pressure level (SPL) for a given time. However, they vary in many ways: time intervals (10 min [6, 12–14], 16 min [15] up to hours [16] or repetitions such as 5 × 45 min [17]), the minimal sound pressure level (from 65 to 80 dB [12, 17, 18]), the distance to the sound level meter (from 2 m [17], 50 cm [19], 40 cm [20] or 30 cm [6, 12, 15], the spectral weighting of sound pressure level (A [6, 21] or C [18]), the type of vocalization (standardized text [13, 19], reading a text of the subject’s choice [17], counting numbers [22], vocalization of vowels [15, 18]), sitting or standing position [17] and whether the minimal SPL was changing in intervals during the test [13, 15, 19] or not [6, 12, 18]. Furthermore, there is no consistency in the analysis of such tests. In some cases pre-post comparisons were performed which analyzed a stroboscopic recording [16, 23], Phonation Threshold Pressure [24, 25], Voice Handicap Index (VHI [26]) [13], acoustic measures [13, 27, 28], visual analog scale [12] or the Dysphonia Severity Index (DSI [29]) [6, 12, 30]. In other examples the material captured during the test itself was analyzed for changes in fundamental frequency (*ƒ*_o_), sound pressure level (SPL) and percentage of SPL below the specified level [31].

In previous studies, it was shown that a vocal loading task of reading a standardized text for 10 min whilst maintaining an SPL of more than 80 dB (A), measured at a distance of 30 cm, caused a drop of the DSI by approximately 0.5–1 point [12, 30]. In addition, it was shown that vocally healthy subjects were able to execute the test without breaks or failures to produce the target SPL [12]. Using accelerometers this vocal loading test also showed vocal doses comparable to teaching a 45-min lesson in a school class [6] which assumes transferability for analysis of realistic professional voice use.

Considering physiological factors, it could be assumed that vocal fatigue by vocal loading is a consequence of vocal fold oscillatory function. However, there are only a few studies analyzing vocal fold oscillatory characteristics with respect to vocal loading. Using high speed videoendoscopy (HSV) Lohscheller et al. [32] analyzed three subjects and Döllinger et al. [33] a single subject by means of phonovibrograms [34]. Both studies found that vocal loading led to changes in the left–right symmetry as well as opening and closing dynamics. However, in both studies, only a very small number of subjects were analyzed and no standardized vocal loading test was used.

The presented study aims to analyze vocal fold oscillation changes after a standardized vocal loading test in vocally healthy subjects using HSV, audio and electroglottographic (EGG) signals. It was hypothesized that vocal fold stiffness would increase due to the loading and, as a consequence, the glottal area waveform (GAW) derived open quotient (OQ) would increase due to vocal fatigue as measured by the DSI. Furthermore, it was hypothesized that such an increase would also be detectable using EGG signals.

## Material and methods

After approval from the local ethical committee. 23 vocally untrained subjects took part (11 female, 12 male, age: 25–45 years), of whom 21 were included in this study. One subject had to be excluded from the dataset because of technical distortions in the audio signal, the other because of distortions in the electroglottographical (EGG) signal. None of the subjects had a medical history of vocal dysfunction or acute voice complaints. All subjects were non-smokers.

All subjects were asked to perform a standardized vocal loading test. Similar to previous studies [12, 30], the subjects were asked to read a standardized text (German Text: Das tapfere Schneiderlein, Grimm brothers) over a 10 min time frame maintaining an SPL higher than 80 dB (A), measured at a distance of 30 cm from the mouth. According to the German Society of Phoniatrics and Pediatric Audiologists the test was not performed in a sound treated environment, but a quasi-living room acoustic setting. The test was performed using the Lingwaves software (Wevosys, Forcheim, Germany). A red arrow on the computer screen was shown when the SPL was lower than the required 80 dB (A). From this vocal loading test, the fundamental frequency (*ƒ*_o_, Hz), SPL [dB (A)] and the shortfall of the 80 dB criterion were calculated as a mean for each minute during the 10-min performance.

Before and after the vocal loading test, a voice range profile (Wevosys, Forchheim, Germany) with a sound level meter (Voltkraft 322, Hong Kong, China) placed at a distance of 30 cm from the mouth was performed. Furthermore, to establish the DSI, also the maximum phonation time (best of three attempts, vowel /a/, comfortable pitch and loudness) was measured and the audio signal from a sustained phonation was recorded for the calculation of the jitter.

Directly before and directly after the vocal loading test rigid high speed video endoscopy (HSV) was performed (HRES-Endocam 5562, Fa. Wolf, Knittlingen, Germany), recorded at 4000 frames per second. For this recording the subject was asked to sustain phonation on the vowel /i/ at comfortable loudness and pitch. Simultaneously, the audio signal using a standard microphone (Sennheiser KE 4–211-1, Sennheiser, Wedemark, Germany, mounted on the laryngoscope at a distance of approximately 10 cm to the lips) and the EGG signal (EG2- PCX2, Glottal Enterprises, Syracuse, USA) were recorded.

For the analysis of the HSV a time window of 1000 frames was segmented using the Glottis Analysis Tools Software (Division of Phoniatrics, University Hospital Erlangen, Germany). From the segmented glottis phonovibrograms were established, as described in detail in Refs. [35, 36]. After this, the Glottal Area Waveform (GAW) as well as the corresponding audio and EGG signal were analyzed using the Multi Signal Analyzer (Division of Phoniatrics, University Hospital Erlangen, Germany). Using this software, numerous numerical data analogous to the Glottal Analysis Tools could be calculated from the different signal types.

For this particular study, the variables are shown in Table 1. For the estimation of the EGG open quotient the Howard criterion [37] was used.

**Table 1:** Computed measures for the three voice signals [audio, glottal area waveform (GAW), electroglottography (EGG)]

Audio	GAW	EGG
Fundamental frequency	Open quotient	Fundamental frequency
Jitter	Closed quotient	Open quotient
PPQ-5	Phase asymmetry index	
RAP	Amplitude symmetry index	
Shimmer	Speed quotient	
APQ-5	Speed index	
HNR	Stiffness	
GNE		

*PPQ *pitch period perturbation quotient, *RAP *relative average peturbation, *APQ *amplitude perturbation quotient, *HNR *harmonic to noise ratio, *GNE *glottal to noise excitation ratio

All statistical analyses were calculated with SPSS 21 (SPSS Inc., Armonk, NY, USA). For the analysis of pre-post differences paired Student’s *t*-tests were used. The level of significance was set to *p* = 0.05.

## Results

**Fig. 1:**
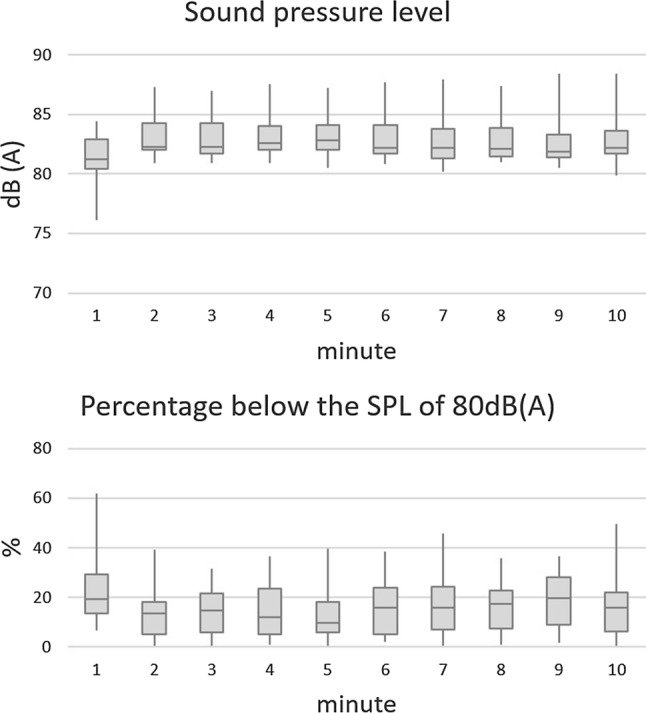
Mean sound pressure level (SPL) and percentage of SPL values below 80 dB (A) for the vocal loading test

**Fig. 2:**
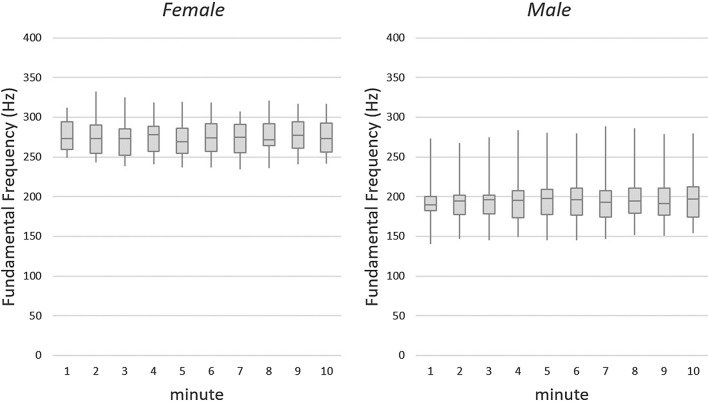
Fundamental frequency for the vocal loading test for the female (left) and male (right) subjects

All subjects completed the vocal loading test without interruption. The results of the vocal loading test for SPL and *ƒ*_o_ for each minute of the test are shown in Fig. 1 and 2.

**Fig. 3:**
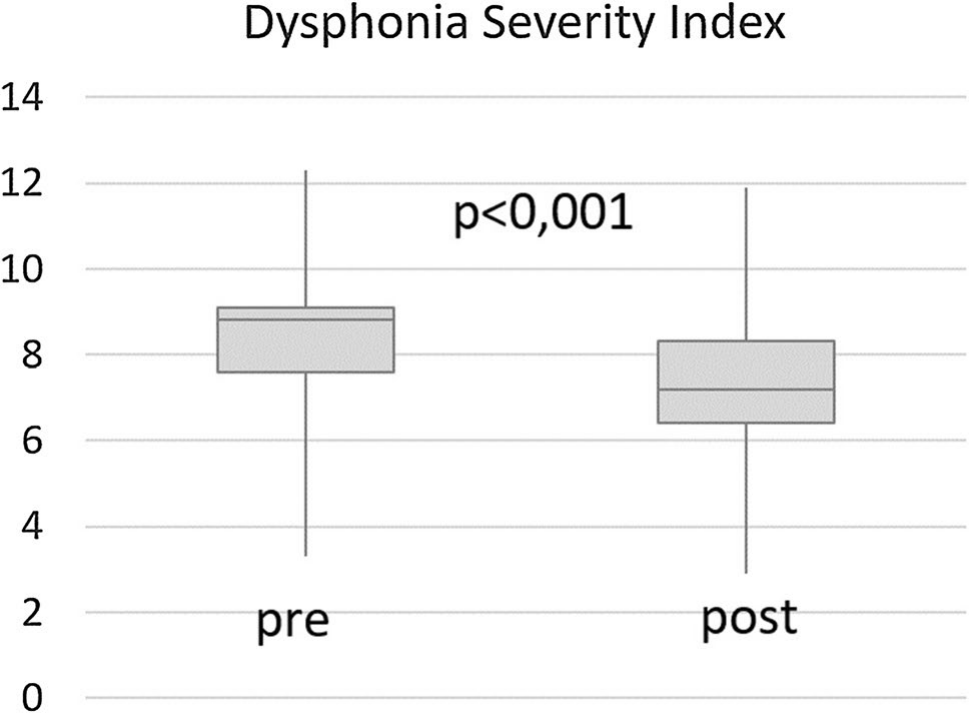
Dysphonia Severity Index before (pre) and after (post) the vocal loading test

In the pre-post loading test comparison, the DSI showed a drop of the mean of 1.18 points (mean 8.34 vs. 7.16, *p* < 0.001, median 8.8 vs.7.2, Fig. 3).

This drop was caused mainly by an increase of the lowest intensity (pre-post comparison for lowest intensity: 45.42 dB vs 48.21 dB, p < 0.01, highest fundamental frequency: 904.10 Hz vs 861.25 Hz, not significant, MPT: 24.21 s vs 22.41 s, not significant, jitter: 0.15% vs 0.15%, not significant). The lowest intensity change was statistically significant for both males and females. Whilst all other factors establishing the DSI failed to show statistically significant changes, the MPT was lowered in the pre-post comparison for males (27.24 s vs. 23.54 s, *p* < 0.05).

In both, the GAW open quotient and the EGG derived open quotient, there was no statistically significant change after the loading test (Table 2). Furthermore, other GAW, EGG and audio signal parameters failed to show any statistically significant change (Table 2).

**Table 2:** Statistical pre-post comparisons (*p* value) for the different variables for the three voice signals [audio, glottal area waveform (GAW), electroglottography (EGG)]

Parameter	*p* value
Audio signal	
Fundamental frequency	> 0.7
Jitter	> 0.3
PPQ-5	> 0.2
RAP	> 0.4
Shimmer	> 0.6
APQ-5	> 0.2
HNR	> 0.9
GNE	> 0.2
Glottal area waveform	
Fundamental frequency	> 0.9
Open quotient	> 0.7
Closed quotient	> 0.7
Phase asymmetry index	> 0.7
Speed quotient	> 0.8
Stiffness	> 0.5
EGG	
Fundamental frequency	> 0.9
Open quotient	> 0.5

*PPQ *pitch period perturbation quotient, *RAP *relative average perturbation, *APQ *amplitude perturbation quotient, *HNR *harmonic to noise ratio, *GNE *glottal to noise excitation ratio

**Fig. 4:**
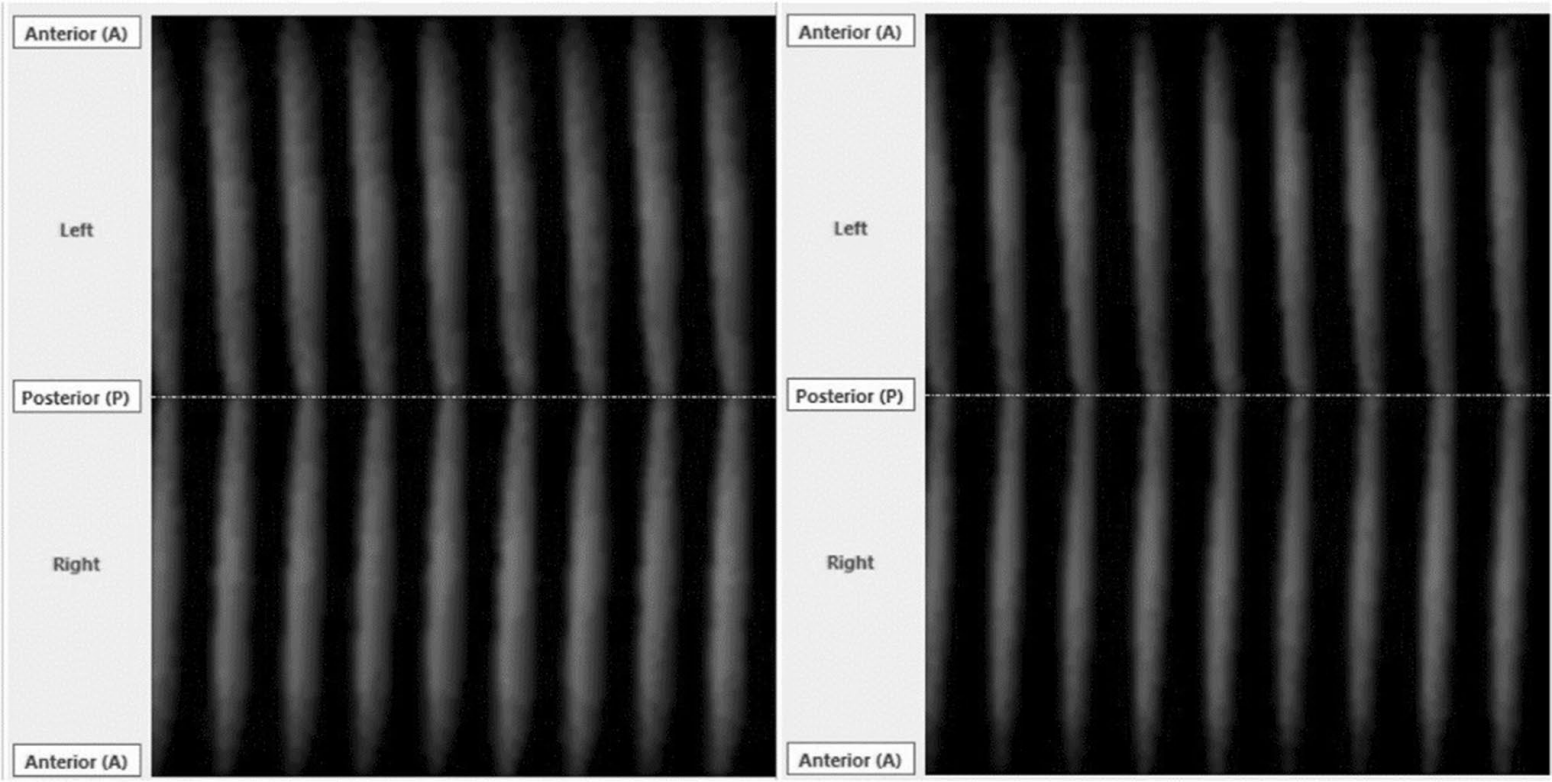
Pre-post comparison of phonovibrogram for subject 2

The oscillatory patterns observed through phonovibrograms were not greatly influenced by the vocal loading. Figure 4 shows a representative phonovibrogram from subject Nr. 2.

There were no statistically significant differences in the pre–post comparison for the Phase Asymmetry Index, Speed Quotient, Speed Index, Stiffness or Amplitude Symmetry.

**Fig. 5:**
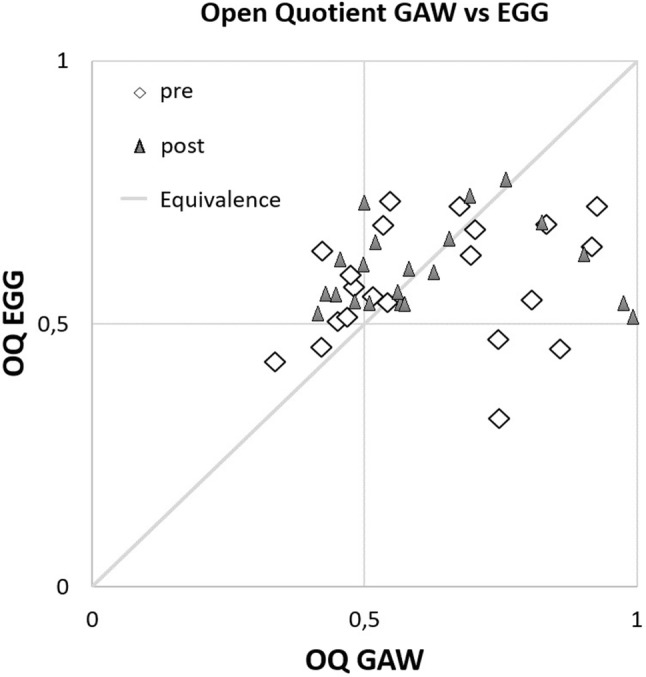
Glottal area waveform derived open quotient (OQ GAW) versus electroglottographic open quotient (OQ EGG)

There was a high level of agreement for the GAW open quotient and the EGG derived open quotient for GAW values lower than 0.7 (trendline: *y* = 0.6183*x* + 0.2727, *r*^2^ = 0.5093), however no correlation for values above this (trendline: *y* = 0.0172*x* + 0.584, *r*^2^ = 0.0003, Fig. 5).

The SPL during the HSV recording, recorded at a distance of 10 cm from the mouth, showed no correlation with the DSI or with the minimum intensity measured at 30 cm from the mouth after the HSV recording.

## Discussion

This study analyses the effects of a standardized vocal loading test on vocal fold oscillation characteristics in vocally healthy subjects. In general, it was found that although the DSI diminished after the loading test, no statistically significant changes were found in the parameters representing vocal fold oscillatory characteristics that were considered.

In previous studies, it has been shown that vocal loading is considered an important indicator of vocal health and that vocal loading can be measured using standardized vocal loading tests [12, 13, 19, 22, 31]. Whereas dysphonic patients show interruptions during the loading [31] or short comings during the loading [19], healthy subjects are usually able to fulfil the requirements of such a test [12, 30]. In the present study, no interruptions or breaks occurred during the test and subjects generally maintained a volume above the target 80 dB (A) criterion. Furthermore, a drop of the DSI was present after the loading, indicating a sign of vocal fatigue. The amount of the drop of 1.2 was only slightly greater than in a previous study [12]. Therefore, it can be concluded that the vocal loading test had an effect on the vocal capacity of the subjects.

However, at the same time there were no statistically significant changes of the oscillatory characteristics measured from the glottal area waveform in the pre-post design. The DSI was mainly caused by a rise of the minimal intensity for both genders and an additional small drop of MPT for the male subjects only. Because subjects were asked only to produce comfortable pitch and loudness, it might have been the case that the loss of soft phonation after the vocal loading test is not reflected in the HSV derived data. The rise of minimal intensity should be related to increased stiffness of the vocal folds with the consequence of increased subglottic pressure and fundamental frequency [38]. However, the HSV data failed to show any statistically significant changes for comfortable pitch and loudness in the pre-post setting. It might be that an increase in stiffness contributes more to vulnerable parts of voice production, such as the phonation threshold pressure which is closely related to the minimum intensity in the voice range profile as has been shown previously [24, 25]. As a consequence, it should be stated that it is necessary to analyze vocal fold oscillations with respect to softest loudness and phonation threshold pressure rather than comfortable pitch and loudness to verify signs of vocal fatigue associated with a vocal loading test. In this context, it should be mentioned that the analysis of the EGG signal with respect to the open quotient might be problematic. As already stated in previous studies [39] the agreement with the GAW-derived open quotient was great up to a value of approximately 0.7. However, above this value the EGG derived open quotient no longer provides valid data.

Furthermore, this discrepancy in results between the DSI and other parameters suggests that comfortable speaking voice is not greatly affected by this standardized loading task. Although signs of fatigue are present by means of the drop of DSI, people are able to continue maintaining their voice at comfortable loudness and pitch. However, it could be speculated that a greater dose would also affect the mean speaking voice. Therefore, it could be of interest for future studies to analyze how much dose would cause modifications of comfortable pitch and loudness. Furthermore, it could be of interest to observe whether greater drops in the DSI could increase the risk of influencing comfortable pitch and loudness.

There are some important limitations of the presented study. This study included vocally healthy subjects only. It cannot be excluded that dysphonic patients would react differently concerning such a standardized vocal loading test. In this respect, it could also be assumed that the overall "effective vocal load" (i.e. effect of multiple parameters) might be different if there is already another vocally taxing factor that is influencing the glottis (e.g. reflux, smoking, allergies, inhalative medications, etc.), even in non-dysphonic subjects. Although other diseases including reflux or pulmonary diseases and smoking history have been excluded by medical history no control of hydration was performed. In a previous study, it has been shown that hydration might impact vocal loading capacity [40]. Furthermore, the loading with 10 min was rather short. Usually, the realistic vocal loading during the day is in voice professionals much greater. It remains unclarified if oscillation patterns at comfortable pitch could be changed in greater doses of vocal loading. Last, HSV was recorded with a rather low sampling rate of 4000 fps. Additionally, it was performed using a rigid endoscopy, which could influence vocal tract shape by a greater amount. It was shown in physiological studies that HSV is possible with a sampling rate of 20,000 fps using flexible endoscopy [41]. The system used in this study, however, had the great advantage of color images, which improved the segmentation process.

## Conclusions

A vocal loading test of text reading at 80 dB(A) or greater measured at 30 cm distance from the mouth influences the dysphonia severity index but not vocal fold oscillations at comfortable pitch in non-dysphonic subjects.
